# The Book World of Medicine and Science

**Published:** 1903-09-19

**Authors:** 


### The Book World of Medicine and Science.

International Clinics. Vol. I. 13th Series. 1903. Edited by A. O. J. Kelly, A.M., M.D. Philadelphia. (London: Lippincott. 1903. 306 pp., with numerous plates and figures. 10s. 6d.)

These "Clinics" deserve to be widely known, for they possess a wealth of information from the pens of some of the most distinguished teachers of medicine at the present time. This volume contains an important contribution by Osler on the general features of the 14 cases of aneurism of the descending thoracic aorta, which represent all the instances of this particular class of aneurism admitted to the wards of the John Hopkins Hospital since its foundation. Though no portion of the aorta is so little subject to aneurism, the latency which is the special character of a certain proportion of cases which may suddenly terminate in death by profuse loss of blood makes the study of the affection peculiarly interesting. In connection with the possible duration of an aneurism he quotes cases in which the symptoms indicated that the disease must have existed 12, 14, 25, and even 30 years before it caused death. In another article Ross describes eight illustrative cases of acquired umbilical hernia in the adult, and Manley makes an important and suggestive observation on the togography of the abdominal organs. He lays stress on the fact that the right side of the abdomen, from a clinical standing-point, teems with interest; for it is on this side we find the greater portion of the liver, the gall-bladder, the portal vein, the pylorus, the head of the pancreas, the duodenum, right kidney, cæcum and appendix, and that portion of the sigmoid loop now known to anatomists as the pelvic portion. Probably 90 per cent. of all serious functional derangements and organic diseases of the abdomen start in the organs situated in the right side. An important lecture, by Thomson, on convulsions in young children follows, and is given in the section on pediatrics, and the important questions which beset practitioners are most clearly and concisely considered. The latter third of the volume is devoted to progress of medicine; and as study and research never cease, this portion is of especial value. Recent statistics on the prevalence of cancer are summarised, showing an undoubted increase in nearly every country in Europe, the United States, and parts of South America. Nowhere does there appear to be any record of decrease. The claims of the adherents to the parasitic theory of cancer are formulated, and to the questions "Is cancer contagious?" and "Does it prevail in certain locations?" instances are recorded which favour the suggestion that answers in the affirmative should be given. Notes are made on nearly all recent important communications in medicine, therapeutics, and surgery, and the subject-matter throughout is equally good. The volumes are published quarterly in pleasing and substantial form, without being in any degree bulky.

Elementary Bacteriology. By M. L. Dhingra, M.D., C.M.Edin. (London: Longmans, Green and Co. 1903. Pp. 145, with illustrations. Price 3s. net.)

The author states in the preface that his aim in writing this little work is to point out principles and suggest problems rather than to marshal facts, and it must be at once conceded that he has very pleasantly achieved his object. The matter is treated in such a way as to be quite easily understood by any intelligent reader even though he have no previous knowledge of bacteriology whatever, and moreover it is dealt with rather from a general standpoint than from that of the practitioner of medicine. Special stress is placed upon those features which are likely to be of importance to medical practitioners and others in India. The book is divided into two parts, and there are two appendices. Part I. deals with various problems connected with the study of bacteria and includes chapters on the theory of spontaneous generation, fermentation, putrefaction, antiseptics and disinfectants, and the preservation of food stuffs. Part II. is devoted to a consideration of the pathogenic bacteria. The two appendices are occupied by descriptions of the principles of bacteriological technique, and snake venom and anti-venomous serum respectively. In the opening chapter on spontaneous generation the author inclines to the belief that such a process is possible, his two main arguments being that, in the first place, the line of demarcation between the organic and the inorganic is being gradually obliterated—for example by the discovery of ferments which occupy an intermediate position between the lifeless proteids and the living cells, and secondly, since the evolution of living matter from non-living matter has occurred once in the history of the world it is conceivable that it may happen again. Part II, which deals specifically with the pathogenic bacteria, is admirable, considering the brevity with which each species is dealt with. The article at the end of the book on snake-poison, though not strictly belonging to the province of bacteriology, is none the less appropriate in a book mainly designed for Indian students, and we are inclined to think that one on rabies might have been added with advantage. The type, paper, and general get-up of the book are very good, and we can strongly recommend it to anyone who is about to take up the study of bacteriology and does not wish to have his views restricted by the four walls of a pathological laboratory.

Studies in Comparative Odontology. By Arthur S. Underwood, M.R.C.S., L.D.S. (London: Baillière, Tindall and Cox. 1903. Pp. 152. Price 5s. net.)

We have not often read a book from which we have derived more pleasure or advantage than we have from this one by Professor Underwood. The author is enthusiastic, and has the knack of imparting his enthusiasm to the reader. The book throughout is quite free from all trace of pedantry, and the freshness of his writing is as valuable as it is rare in a work of this kind. We quote the following as an example of Professor Underwood's manner of placing his subject before the reader; he is discussing the teeth of the extinct carnivore smilodon, an animal which had two huge canines in the upper jaw:—"For a long time it was supposed that this creature's upper canines were so long that he could never open his mouth wide enough to use them, and this grotesque idea has actually been elaborated into the quaint suggestion that smilodon and the machœrodonts generally became extinct because they *were literally handicapped out of existence by their huge tusks!* Some writers have suggested that they used the tusks with their mouths shut, others that they employed them for climbing trees, and others that they were aquatic creatures and used these weapons after the manner of dinotherium. There is no reason to suppose they were aquatic; moreover, the sharp points and razor-like posterior edge of these canines would have led to many an awkward tumble in any arboreal exploits." He goes on to show that smilodon could open his mouth wide enough to use his canines to some purpose as weapons of offence. There are numerous illustrations, which are a great help towards thoroughly understanding the text. We believe that the book fills a distinct want, and will be widely read, not only by students of zoology and dentistry, but by everyone who is at all interested in the subject of comparative morphology.

